# Crystal structure of poly[[μ_2_-di­aqua-di­aqua-μ_2_-l-proline-κ^2^ *O*:*O*′-strontium] dibromide]

**DOI:** 10.1107/S2056989015017302

**Published:** 2015-09-26

**Authors:** Selladurai Sathiskumar, Thangavelu Balakrishnan, Kandasamy Ramamurthi, Subbiah Thamotharan

**Affiliations:** a Crystal Growth Laboratory, PG and Research Department of Physics, Periyar EVR College (Autonomous), Tiruchirappalli 620 023, India; b Crystal Growth and Thin Film Laboratory, Department of Physics and Nanotechnology, SRM University, Kattankulathur 603 203, India; c Biomolecular Crystallography Laboratory, Department of Bioinformatics, School of Chemical and Biotechnology, SASTRA University, Thanjavur 613 401, India

**Keywords:** crystal structure, proline, amino acid, strontium coordination polymer, N/O—H⋯Br hydrogen bonds

## Abstract

In the title coordination polymer, {[Sr(C_5_H_9_NO_2_)(H_2_O)_4_]Br_2_}_*n*_, the proline mol­ecule exists in a zwitterionic form with one of the ring C atoms disordered over two sites [site-occupancy factors = 0.57 (6):0.43 (6)]. The Sr^II^ ion is nine-coordinated by six water O atoms, two monodentate and two μ_2_-bridging, and three carboxyl­ate O atoms of the proline ligands, with two bridging [Sr—O range = 2.524 (4)–2.800 (5) Å]. In the crystal, there is no direct inter­action between the proline mol­ecules. However, the proline and water mol­ecules associate with the bromide counter-anions through a number of inter­molecular O—H⋯Br and N—H⋯Br hydrogen-bonding inter­actions, giving a three-dimensional supra­molecular structure.

## Abstract 2

In the title polymer, zwitterionic proline and water mol­ecules inter­act with the bromide counter-anions through inter­molecular N—H⋯Br and O—H⋯Br hydrogen-bonding inter­actions, providing a novel supra­molecular structure.

## Chemical context   

The study of coordination polymers has been an area of rapid development in recent years due to their inter­esting structures and their wide range of applications as functional materials (Lyhs *et al.*, 2012 ▸). Reports of the crystal structures of alkaline earth metal ions combined with anions of amino acids are very limited. As part of our ongoing investigations of the crystal and mol­ecular structures of a series of metal complexes generated from amino acids (Revathi *et al.*, 2015 ▸; Sathiskumar *et al.*, 2015**a* ▸,b* ▸; Balakrishnan *et al.*, 2013 ▸), we report here the crystal structure of a polymeric strontium–proline complex, {[Sr(C_5_H_9_NO_2_)(H_2_O)_4_]^2+^ 2(Br^−^)}_*n*_, (I).
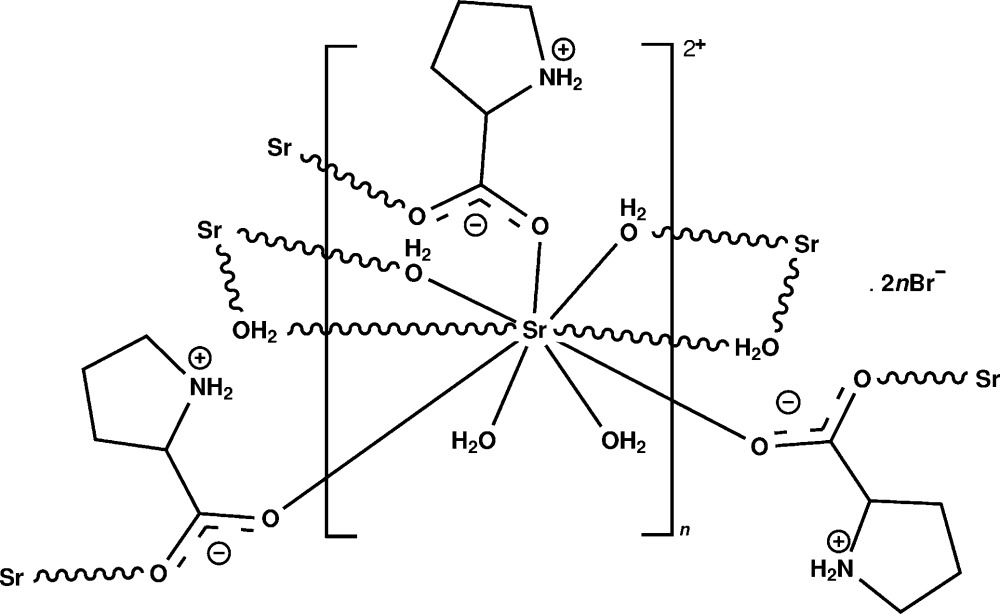


## Structural commentary   

**Figure 1:**
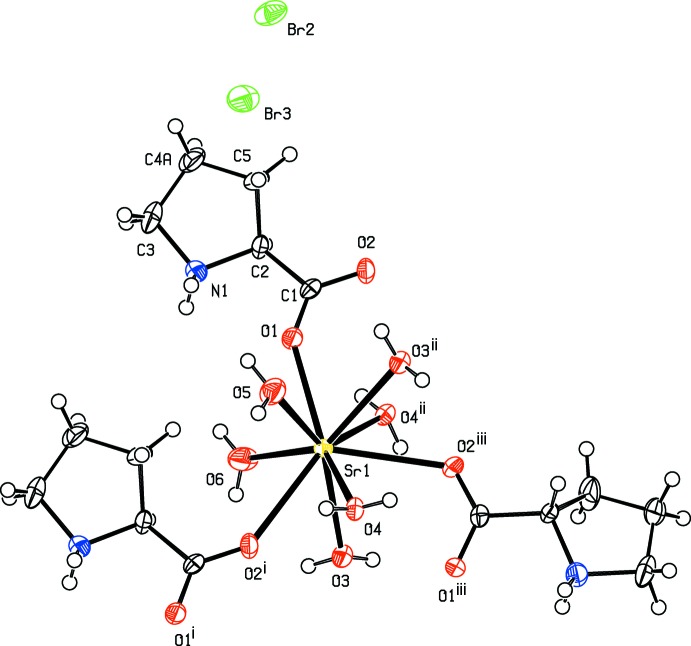
The coordination sphere of Sr^2+^ in the crystal structure of (I). Only the major components of the disordered proline ligands are shown. Displacement ellipsoids are drawn at the 50% probability level. For symmetry codes, see Table 1 ▸.

**Figure 2:**
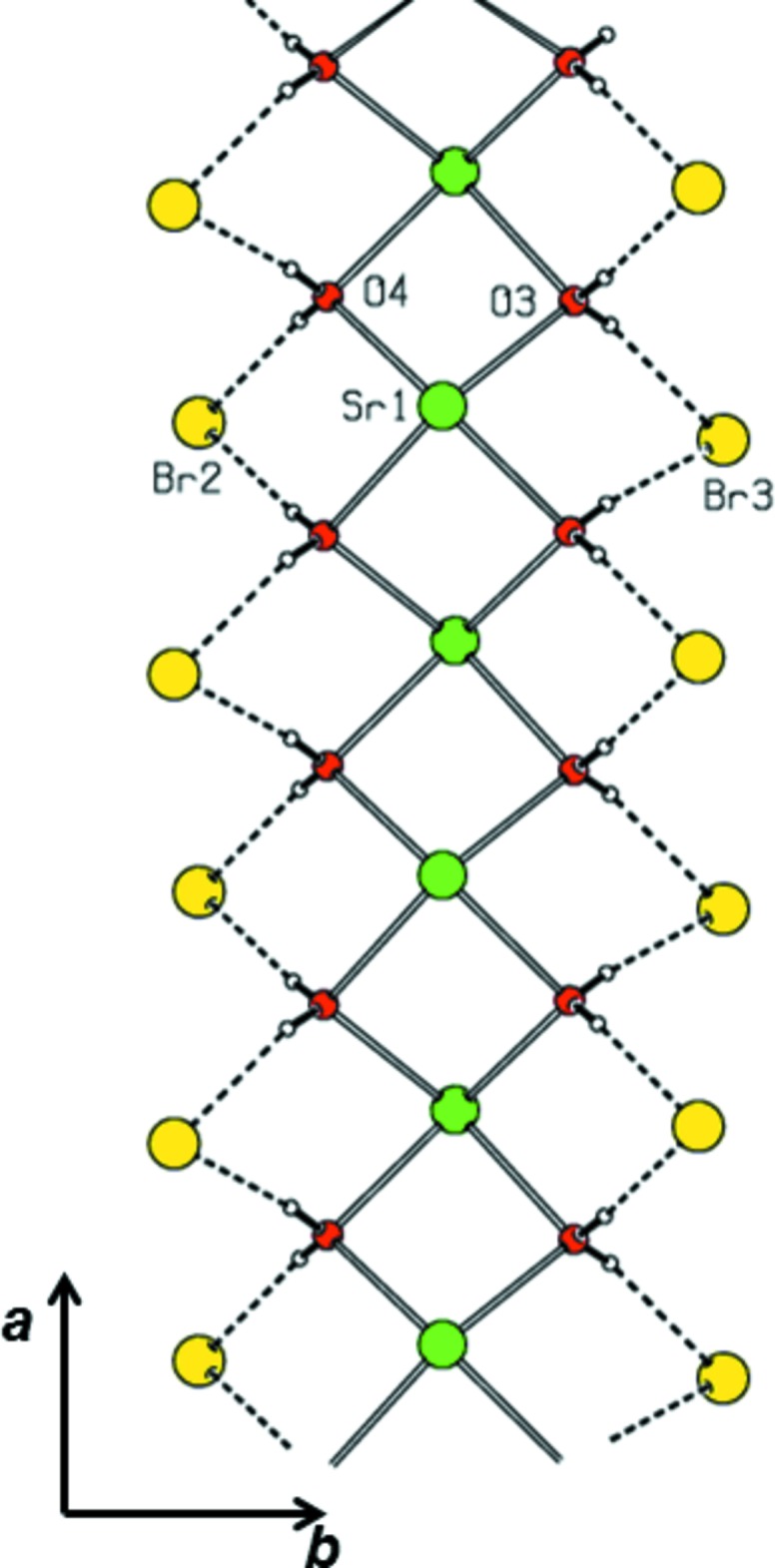
The Sr–water coordination polymeric chain substructure of (I), with peripheral water O—H⋯Br hydrogen bonds shown as dashed lines.

The asymmetric unit of the title complex (I) contains one Sr^2+^ ion, one bridging proline ligand and four water mol­ecules, two of which are monodentate and two bridging, and two bromide counter-anions (Fig. 1 ▸). In (I), the bond lengths involving the carboxyl­ate atoms and the protonation of the amino group suggest that the proline mol­ecule exists in a zwitterionic form. The Sr^II^ ion is nine-coordinated by six water oxygen atoms [Sr—O = 2.582 (6)–2.707 (5)Å] and three carboxyl­ate oxygen atoms of zwitterionic proline ligands [Sr—O = 2.524 (4)–2.800 (4) Å; Table 1 ▸]. In the strontium–glycine complex, the Sr—O (water) and Sr—O(carboxyl­ate) distances ranges are 2.526 (4)–2.661 (2) and 2.605 (2)–2.703 (2) Å, respectively (Revathi *et al.*, 2015 ▸). In (I), one of the carbon atoms (C4) of the pyrrolidine ring is disordered over two sites. In the major component of the pyrrolidine ring, there is a twist conformation on the C2—C5 bond with a pseudo-rotation angle Δ = 40.1 (14)° and a maximum torsion angle φ_m_ = 43.8 (10)° for the atom sequence N1–C2–C5–C4*A*–C3 (Rao *et al.*, 1981 ▸). In the minor component, the pyrrolidine ring exhibits an envelope conformation on N1 with a pseudo-rotation angle Δ = 341.5 (19)° and a maximum torsion angle φ_m_ = 36.0 (9)° for the atom sequence N1–C2–C5–C4*B*–C3 (Rao *et al.*, 1981 ▸). As shown in Fig. 2 ▸, the title complex forms a coordination polymeric chain that lies parallel to the *a* axis. Adjacent Sr^II^ ions are separated by 3.9387 (7) Å within a chain.

## Supra­molecular features   

The crystal structure of (I), is stabilized by inter­molecular N—H⋯Br and O—H⋯Br hydrogen bonds (Table 2 ▸). One of the characteristic features observed in amino acid complexes is the head-to-tail sequence in which amino acids are self-associated through their amino and carboxyl­ate groups (Sharma *et al.*, 2006 ▸; Selvaraj *et al.*, 2007 ▸; Balakrishnan *et al.*, 2013 ▸; Revathi *et al.*, 2015 ▸). In the crystal structure of the l-proline lithium bromide monohydrate complex, there is a head-to-tail sequence observed (Sathiskumar *et al.*, 2015*a* ▸). In contrast, there is no direct hydrogen-bonding inter­action between the proline mol­ecules in (I).

**Figure 3:**
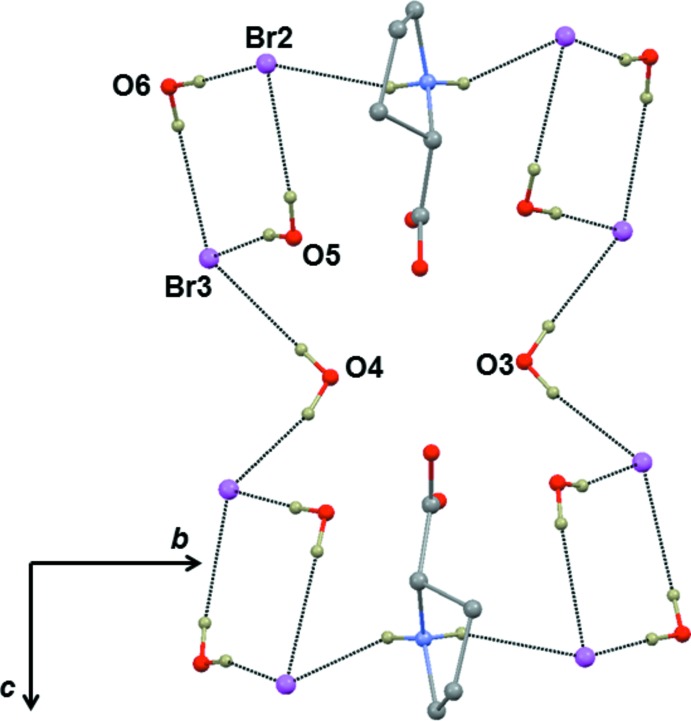
The butterfly-like supra­molecular arrangements generated by inter­molecular N—H⋯Br and O—H⋯Br hydrogen bonds. Only atoms involved in hydrogen-bonding inter­actions are labelled.

**Figure 4:**
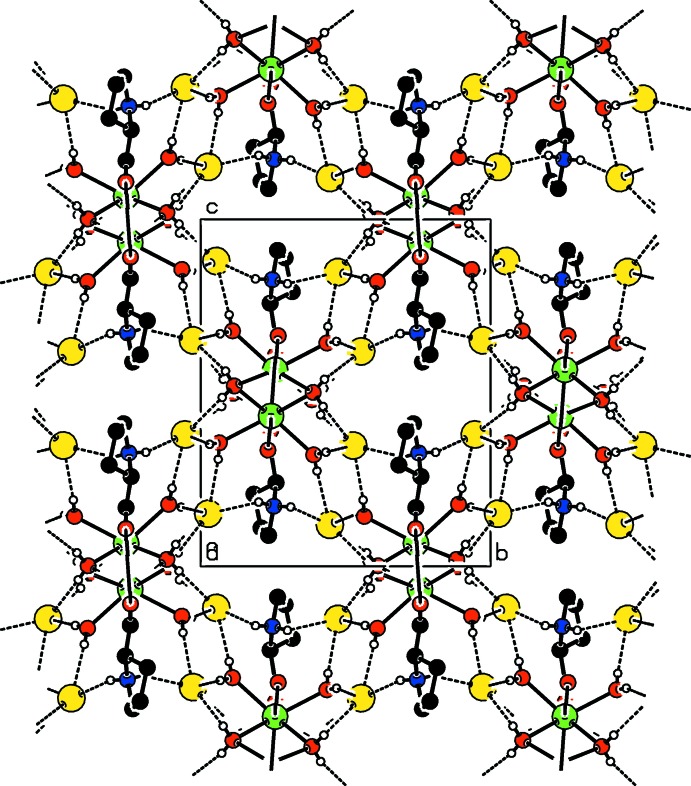
The crystal packing of (I) viewed along the *a* axis, with hydrogen bonds shown as dashed lines. C-bound H atoms have been omitted for clarity.

As shown in Fig. 3 ▸, two water mol­ecules and two bromide anions along with Sr^2+^ ions generate a hydrogen-bonded sheet which lies parallel to the *a* axis. Within this sheet, two Sr^2+^ ions and two water oxygens form a cyclic motif. Water mol­ecules (O3 and O4) inter­connect the bromide anions, forming a chain. In (I), two mol­ecules (O5 and O6) act as donors for inter­molecular O—H⋯Br hydrogen bonds. These hydrogen bonds generate a cyclic dibromide motif similar to that observed in a related structure (Revathi *et al.*, 2015 ▸). Adjacent dibromide motifs in (I), which run parallel to the *b* axis, are inter­connected by proline ligands through inter­molecular N—H⋯Br hydrogen bonds on both sides (Fig. 3 ▸). Adjacent supra­molecular arrangements of cyclic dibromide⋯proline⋯cyclic dibromide motifs are inter­linked further by water mol­ecules (O3 and O4) through O—H⋯Br hydrogen bonds. This entire arrangement forms a butterfly-like structure. The overall hydrogen-bonded supra­molecular structure (Fig. 4 ▸) is three-dimensional.

## Synthesis and crystallization   

Single crystals of the title complex were obtained by slow evaporation from an aqueous solution of l-proline and strontium bromide hexa­hydrate in a 1:1 stoichiometric molar ratio at 306 K. The prepared solution was stirred well and filtered. The resultant filtered solution was left undisturbed to allow evaporation. After 15 days, colourless prismatic crystals were harvested.

## Refinement   

Crystal data, data collection and structure refinement details are summarized in Table 3 ▸. One of the carbon (C4) atoms of the pyrrolidine ring appears to be disordered over two sites. These positions were defined for this atom and constrained refinement of the site-occupation factors led to a value of 0.57 (6) for the major component. The positions of amino and water H atoms were located from difference Fourier maps. Further, the O—H distances in the water mol­ecules were restrained to 0.85 (2) Å. The N—H distances of amino group were also restrained, to 0.89 (2) Å. The remaining hydrogen atoms were placed in geometrically idealized positions (C—H = 0.97 Å with *U*_iso_(H) = 1.2*U*_eq_(C) and were constrained to ride on their parent atom. The Flack absolute structure parameter was determined to be 0.008 (8) (788 Friedel pairs; Parsons *et al.*, 2013 ▸), indicating an *S* configuration for C2, consistent with that for the parent l-proline (Kayushina & Vainshtein, 1965 ▸).

## Supplementary Material

Crystal structure: contains datablock(s) I. DOI: 10.1107/S2056989015017302/zs2346sup1.cif

Structure factors: contains datablock(s) I. DOI: 10.1107/S2056989015017302/zs2346Isup2.hkl

CCDC reference: 1424731

Additional supporting information:  crystallographic information; 3D view; checkCIF report

## Figures and Tables

**Table 1:** Selected bond lengths ()

Sr1O1	2.524(4)	Sr1O2^i^	2.728(4)
Sr1O3	2.625(6)	Sr1O3^ii^	2.707(6)
Sr1O4	2.630(6)	Sr1O4^ii^	2.651(5)
Sr1O5	2.593(5)	Sr1O2^iii^	2.800(5)
Sr1O6	2.582(6)		

Symmetry codes: (i) 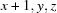; (ii) 
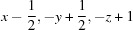
; (iii) 
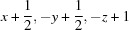
.

**Table 2:** Hydrogen-bond geometry (, )

*D*H*A*	*D*H	H*A*	*D* *A*	*D*H*A*
N1H1*A*Br2^i^	0.90(6)	2.52(5)	3.374(7)	159(6)
N1H1*B*Br3^i^	0.90(7)	2.40(7)	3.240(7)	156(8)
O3H3*C*Br3^iv^	0.84(7)	2.63(7)	3.440(6)	163(7)
O3H3*D*Br2^v^	0.84(7)	2.54(7)	3.376(6)	172(5)
O4H4*E*Br2^vi^	0.85(6)	2.47(7)	3.281(6)	162(7)
O4H4*F*Br3^vii^	0.83(6)	2.52(6)	3.347(6)	174(6)
O5H5*C*Br2^i^	0.86(5)	2.54(5)	3.369(6)	164(6)
O5H5*D*Br3^vii^	0.84(6)	2.48(6)	3.304(6)	166(6)
O6H6*C*Br2^v^	0.83(6)	2.58(6)	3.393(6)	167(5)
O6H6*D*Br3^i^	0.85(7)	2.56(6)	3.378(6)	162(7)

Symmetry codes: (i) 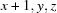; (iv) 
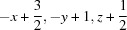
; (v) 
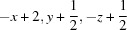
; (vi) 
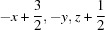
; (vii) 
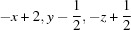
.

**Table 3:** Experimental details

Crystal data	
Chemical formula	[Sr(C_5_H_9_NO_2_)(H_2_O)_4_]Br_2_
*M*_r_	434.63
Crystal system, space group	Orthorhombic, *P*2_1_2_1_2_1_
Temperature (K)	296
*a*, *b*, *c* ()	6.7079(4), 12.9125(9), 15.4499(11)
*V* (^3^)	1338.20(16)
*Z*	4
Radiation type	Mo *K*
(mm^1^)	10.01
Crystal size (mm)	0.15 0.10 0.10

Data collection	
Diffractometer	Bruker Kappa APEXII CCD
Absorption correction	Multi-scan (*SABABS*; Bruker, 2004 ▸)
*T*_min_, *T*_max_	0.26, 0.44
No. of measured, independent and observed [*I* > 2(*I*)] reflections	14183, 2345, 2081
*R*_int_	0.068
(sin /)_max_ (^1^)	0.594

Refinement	
*R*[*F* ^2^ > 2(*F* ^2^)], *wR*(*F* ^2^), *S*	0.032, 0.063, 1.07
No. of reflections	2345
No. of parameters	186
No. of restraints	26
H-atom treatment	H atoms treated by a mixture of independent and constrained refinement
_max_, _min_ (e ^3^)	0.60, 0.86
Absolute structure	Flack *x* determined using 788 quotients [(*I* ^+^)(*I* )]/[(*I* ^+^)+(*I* )] (Parsons *et al.*, 2013 ▸)
Absolute structure parameter	0.008(8)

Computer programs: *APEX2*, *SAINT* and *XPREP* (Bruker, 2004 ▸), *SIR92* (Altomare *et al.*, 1993 ▸), *SHELXL2014* (Sheldrick, 2015 ▸), *PLATON* (Spek, 2009 ▸) and *Mercury* (Macrae *et al.*, 2008 ▸).

## supplementary crystallographic information

### Crystal data

**Table:**

[Sr(C_5_H_9_NO_2_)(H_2_O)_4_]Br_2_	*D*_x_ = 2.157 Mg m^−^^3^
*M**_r_* = 434.63	Mo *K*α radiation, λ = 0.71073 Å
Orthorhombic, *P*2_1_2_1_2_1_	Cell parameters from 7063 reflections
*a* = 6.7079 (4) Å	θ = 2.6–28.5°
*b* = 12.9125 (9) Å	µ = 10.01 mm^−^^1^
*c* = 15.4499 (11) Å	*T* = 296 K
*V* = 1338.20 (16) Å^3^	Block, brown
*Z* = 4	0.15 × 0.10 × 0.10 mm
*F*(000) = 840	

### Data collection

**Table:**

Bruker Kappa APEXII CCD diffractometer	2081 reflections with *I* > 2σ(*I*)
Radiation source: Sealed tube	*R*_int_ = 0.068
ω nd φ scan	θ_max_ = 25.0°, θ_min_ = 2.6°
Absorption correction: multi-scan (SABABS; Bruker, 2004)	*h* = −7→7
*T*_min_ = 0.26, *T*_max_ = 0.44	*k* = −15→15
14183 measured reflections	*l* = −18→18
2345 independent reflections	

### Refinement

**Table:**

Refinement on *F*^2^	Hydrogen site location: mixed
Least-squares matrix: full	H atoms treated by a mixture of independent and constrained refinement
*R*[*F*^2^ > 2σ(*F*^2^)] = 0.032	*w* = 1/[σ^2^(*F*_o_^2^) + (0.0267*P*)^2^] where *P* = (*F*_o_^2^ + 2*F*_c_^2^)/3
*wR*(*F*^2^) = 0.063	(Δ/σ)_max_ < 0.001
*S* = 1.07	Δρ_max_ = 0.60 e Å^−^^3^
2345 reflections	Δρ_min_ = −0.86 e Å^−^^3^
186 parameters	Absolute structure: Flack *x* determined using 788 quotients [(*I*^+^)-(*I*^-^)]/[(*I*^+^)+(*I*^-^)] (Parsons *et al.*, 2013)
26 restraints	Absolute structure parameter: 0.008 (8)

### Special details

**Table:**

Geometry. Bond distances, angles etc. have been calculated using the rounded fractional coordinates. All su's are estimated from the variances of the (full) variance-covariance matrix. The cell esds are taken into account in the estimation of distances, angles and torsion angles

### Fractional atomic coordinates and isotropic or equivalent isotropic displacement parameters (Å^2^)

**Table:**

	*x*	*y*	*z*	*U*_iso_*/*U*_eq_	Occ. (<1)
Sr1	1.34882 (8)	0.24302 (5)	0.43342 (4)	0.0174 (2)	
O1	1.0492 (6)	0.2322 (4)	0.3350 (3)	0.0257 (16)	
O2	0.7439 (6)	0.2420 (4)	0.3916 (3)	0.0243 (16)	
O3	1.5725 (8)	0.3885 (5)	0.5016 (4)	0.0243 (19)	
O4	1.5819 (8)	0.1162 (4)	0.5200 (4)	0.0213 (17)	
O5	1.3443 (9)	0.0648 (4)	0.3561 (4)	0.035 (2)	
O6	1.3860 (9)	0.3899 (5)	0.3212 (4)	0.038 (2)	
N1	0.9404 (8)	0.2529 (6)	0.1734 (4)	0.026 (2)	
C1	0.8660 (9)	0.2426 (5)	0.3307 (4)	0.018 (2)	
C2	0.7837 (9)	0.2600 (6)	0.2411 (4)	0.021 (2)	
C3	0.8370 (12)	0.2347 (7)	0.0890 (5)	0.042 (3)	
C4A	0.623 (2)	0.211 (3)	0.1117 (11)	0.034 (7)	0.57 (6)
C5	0.6277 (12)	0.1840 (7)	0.2082 (5)	0.042 (3)	
C4B	0.660 (5)	0.167 (3)	0.1117 (13)	0.035 (8)	0.43 (6)
Br2	0.18627 (12)	0.02641 (6)	0.15165 (6)	0.0389 (3)	
Br3	0.22307 (13)	0.44596 (7)	0.11973 (6)	0.0466 (3)	
H1A	1.024 (9)	0.199 (4)	0.180 (5)	0.02 (2)*	
H1B	1.033 (10)	0.303 (5)	0.175 (6)	0.05 (3)*	
H3C	1.522 (13)	0.429 (5)	0.538 (4)	0.06 (3)*	
H3D	1.622 (13)	0.422 (5)	0.460 (4)	0.07 (4)*	
H4E	1.532 (12)	0.085 (5)	0.563 (3)	0.05 (3)*	
H4F	1.640 (10)	0.075 (4)	0.487 (4)	0.04 (3)*	
H5C	1.281 (10)	0.060 (6)	0.308 (3)	0.06 (3)*	
H5D	1.450 (7)	0.030 (6)	0.353 (5)	0.06 (3)*	
H6C	1.478 (8)	0.432 (5)	0.327 (5)	0.03 (3)*	
H6D	1.319 (11)	0.402 (7)	0.276 (4)	0.09 (4)*	
H31	0.89720	0.17700	0.05850	0.0500*	0.57 (6)
H32	0.84480	0.29580	0.05270	0.0500*	0.57 (6)
H41	0.57440	0.15290	0.07800	0.0410*	0.57 (6)
H42	0.53890	0.27050	0.10120	0.0410*	0.57 (6)
H51	0.49920	0.19560	0.23530	0.0500*	0.57 (6)
H52	0.66830	0.11280	0.21770	0.0500*	0.57 (6)
H2	0.72630	0.32970	0.23900	0.0250*	
H33	0.92450	0.19970	0.04850	0.0500*	0.43 (6)
H34	0.79360	0.29960	0.06370	0.0500*	0.43 (6)
H43	0.68850	0.09490	0.09960	0.0420*	0.43 (6)
H44	0.54310	0.18790	0.07910	0.0420*	0.43 (6)
H53	0.49510	0.21140	0.21840	0.0500*	0.43 (6)
H54	0.63970	0.11870	0.23880	0.0500*	0.43 (6)

### Atomic displacement parameters (Å^2^)

**Table:**

	*U*^11^	*U*^22^	*U*^33^	*U*^12^	*U*^13^	*U*^23^
Sr1	0.0142 (3)	0.0215 (4)	0.0165 (3)	0.0003 (3)	−0.0010 (3)	0.0006 (3)
O1	0.017 (2)	0.041 (3)	0.019 (3)	0.006 (2)	−0.001 (2)	−0.003 (3)
O2	0.024 (2)	0.033 (3)	0.016 (3)	0.004 (3)	0.004 (2)	0.002 (3)
O3	0.025 (3)	0.029 (3)	0.019 (4)	0.000 (2)	0.000 (3)	−0.003 (3)
O4	0.024 (3)	0.024 (3)	0.016 (3)	0.000 (2)	0.003 (3)	0.000 (3)
O5	0.033 (3)	0.044 (4)	0.029 (4)	0.006 (3)	−0.002 (3)	−0.010 (3)
O6	0.034 (4)	0.043 (4)	0.037 (4)	−0.012 (3)	−0.011 (3)	0.017 (3)
N1	0.022 (3)	0.035 (4)	0.022 (4)	−0.005 (4)	0.000 (3)	0.004 (4)
C1	0.025 (4)	0.011 (3)	0.017 (4)	−0.001 (3)	−0.003 (3)	−0.004 (3)
C2	0.024 (3)	0.026 (4)	0.013 (4)	0.005 (4)	0.002 (3)	0.000 (4)
C3	0.052 (5)	0.059 (6)	0.015 (4)	0.000 (5)	−0.001 (4)	−0.001 (4)
C4A	0.036 (10)	0.041 (15)	0.025 (9)	0.001 (8)	−0.007 (7)	−0.014 (9)
C5	0.035 (5)	0.066 (6)	0.025 (5)	−0.025 (4)	−0.006 (4)	−0.004 (5)
C4B	0.033 (13)	0.042 (17)	0.030 (11)	−0.010 (13)	0.005 (10)	−0.023 (11)
Br2	0.0357 (5)	0.0379 (5)	0.0432 (5)	0.0057 (4)	−0.0102 (4)	−0.0197 (4)
Br3	0.0505 (6)	0.0484 (6)	0.0409 (6)	−0.0239 (4)	−0.0107 (4)	0.0197 (5)

### Geometric parameters (Å, º)

**Table:**

Sr1—O1	2.524 (4)	N1—H1A	0.90 (6)
Sr1—O3	2.625 (6)	N1—H1B	0.90 (7)
Sr1—O4	2.630 (6)	C1—C2	1.507 (9)
Sr1—O5	2.593 (5)	C2—C5	1.522 (11)
Sr1—O6	2.582 (6)	C3—C4A	1.509 (17)
Sr1—O2^i^	2.728 (4)	C3—C4B	1.52 (4)
Sr1—O3^ii^	2.707 (6)	C4A—C5	1.53 (2)
Sr1—O4^ii^	2.651 (5)	C4B—C5	1.52 (2)
Sr1—O2^iii^	2.800 (5)	C2—H2	0.9800
O1—C1	1.238 (7)	C3—H31	0.9700
O2—C1	1.248 (8)	C3—H32	0.9700
O3—H3C	0.84 (7)	C3—H33	0.9700
O3—H3D	0.84 (7)	C3—H34	0.9700
O4—H4E	0.85 (6)	C4A—H41	0.9700
O4—H4F	0.83 (6)	C4A—H42	0.9700
O5—H5D	0.84 (6)	C4B—H43	0.9700
O5—H5C	0.86 (5)	C4B—H44	0.9700
O6—H6D	0.85 (7)	C5—H51	0.9700
O6—H6C	0.83 (6)	C5—H54	0.9700
N1—C3	1.496 (10)	C5—H52	0.9700
N1—C2	1.486 (8)	C5—H53	0.9700
			
O1—Sr1—O3	137.44 (18)	Sr1—O6—H6D	130 (6)
O1—Sr1—O4	138.19 (17)	Sr1—O6—H6C	119 (5)
O1—Sr1—O5	70.37 (18)	H6C—O6—H6D	111 (8)
O1—Sr1—O6	73.32 (18)	C2—N1—C3	107.2 (5)
O1—Sr1—O2^i^	129.10 (14)	C3—N1—H1B	117 (6)
O1—Sr1—O3^ii^	69.11 (17)	H1A—N1—H1B	97 (5)
O1—Sr1—O4^ii^	70.35 (17)	C2—N1—H1A	114 (5)
O1—Sr1—O2^iii^	112.66 (13)	C2—N1—H1B	115 (5)
O3—Sr1—O4	84.36 (18)	C3—N1—H1A	105 (5)
O3—Sr1—O5	145.76 (18)	O1—C1—C2	115.4 (5)
O3—Sr1—O6	71.85 (19)	O2—C1—C2	117.0 (5)
O2^i^—Sr1—O3	62.82 (16)	O1—C1—O2	127.6 (6)
O3—Sr1—O3^ii^	133.75 (19)	N1—C2—C5	102.2 (6)
O3—Sr1—O4^ii^	77.67 (17)	N1—C2—C1	112.2 (5)
O2^iii^—Sr1—O3	72.95 (16)	C1—C2—C5	117.6 (6)
O4—Sr1—O5	71.86 (18)	N1—C3—C4B	104.6 (10)
O4—Sr1—O6	137.87 (18)	N1—C3—C4A	105.7 (8)
O2^i^—Sr1—O4	62.60 (16)	C3—C4A—C5	104.7 (10)
O3^ii^—Sr1—O4	80.08 (17)	C3—C4B—C5	104.8 (19)
O4—Sr1—O4^ii^	133.67 (19)	C2—C5—C4A	101.1 (12)
O2^iii^—Sr1—O4	72.62 (16)	C2—C5—C4B	108.8 (15)
O5—Sr1—O6	110.10 (19)	N1—C2—H2	108.00
O2^i^—Sr1—O5	84.14 (17)	C1—C2—H2	108.00
O3^ii^—Sr1—O5	66.80 (19)	C5—C2—H2	108.00
O4^ii^—Sr1—O5	136.53 (18)	N1—C3—H31	111.00
O2^iii^—Sr1—O5	120.21 (17)	N1—C3—H32	111.00
O2^i^—Sr1—O6	75.56 (17)	N1—C3—H33	111.00
O3^ii^—Sr1—O6	140.90 (18)	N1—C3—H34	111.00
O4^ii^—Sr1—O6	75.16 (19)	C4A—C3—H31	111.00
O2^iii^—Sr1—O6	128.45 (18)	C4A—C3—H32	111.00
O2^i^—Sr1—O3^ii^	138.60 (17)	H31—C3—H32	109.00
O2^i^—Sr1—O4^ii^	136.37 (16)	C4B—C3—H33	111.00
O2^i^—Sr1—O2^iii^	118.24 (13)	C4B—C3—H34	111.00
O3^ii^—Sr1—O4^ii^	82.36 (17)	H33—C3—H34	109.00
O2^iii^—Sr1—O3^ii^	60.87 (16)	C3—C4A—H41	111.00
O2^iii^—Sr1—O4^ii^	61.37 (16)	C3—C4A—H42	111.00
Sr1—O1—C1	144.8 (4)	C5—C4A—H41	111.00
Sr1^iv^—O2—C1	144.7 (4)	C5—C4A—H42	111.00
Sr1^ii^—O2—C1	124.2 (4)	H41—C4A—H42	109.00
Sr1^iv^—O2—Sr1^ii^	90.87 (13)	H43—C4B—H44	109.00
Sr1—O3—Sr1^iii^	95.2 (2)	C3—C4B—H44	111.00
Sr1—O4—Sr1^iii^	96.47 (17)	C5—C4B—H43	111.00
H3C—O3—H3D	111 (6)	C3—C4B—H43	111.00
Sr1^iii^—O3—H3C	115 (5)	C5—C4B—H44	111.00
Sr1^iii^—O3—H3D	110 (5)	C2—C5—H52	112.00
Sr1—O3—H3C	119 (6)	C2—C5—H53	110.00
Sr1—O3—H3D	107 (5)	C2—C5—H51	112.00
H4E—O4—H4F	111 (6)	C4B—C5—H54	110.00
Sr1—O4—H4E	117 (5)	H53—C5—H54	108.00
Sr1—O4—H4F	111 (4)	C2—C5—H54	110.00
Sr1^iii^—O4—H4F	107 (4)	C4A—C5—H51	112.00
Sr1^iii^—O4—H4E	112 (5)	C4A—C5—H52	112.00
Sr1—O5—H5D	119 (5)	H51—C5—H52	109.00
Sr1—O5—H5C	118 (5)	C4B—C5—H53	110.00
H5C—O5—H5D	109 (7)		
			
O3—Sr1—O1—C1	−76.6 (8)	O5^iii^—Sr1^iii^—O3—Sr1	−158.1 (2)
O4—Sr1—O1—C1	101.4 (8)	O6^iii^—Sr1^iii^—O3—Sr1	−64.4 (3)
O5—Sr1—O1—C1	128.0 (8)	O1—Sr1—O4—Sr1^iii^	171.61 (16)
O6—Sr1—O1—C1	−112.8 (8)	O3—Sr1—O4—Sr1^iii^	−9.71 (18)
O2^i^—Sr1—O1—C1	−167.5 (7)	O5—Sr1—O4—Sr1^iii^	145.3 (2)
O3^ii^—Sr1—O1—C1	56.1 (8)	O6—Sr1—O4—Sr1^iii^	45.1 (3)
O4^ii^—Sr1—O1—C1	−33.0 (8)	O2^i^—Sr1—O4—Sr1^iii^	52.53 (16)
O2^iii^—Sr1—O1—C1	12.5 (8)	O3^ii^—Sr1—O4—Sr1^iii^	−146.0 (2)
O1^iv^—Sr1^iv^—O2—C1	5.3 (8)	O4^ii^—Sr1—O4—Sr1^iii^	−76.7 (3)
O3^iv^—Sr1^iv^—O2—C1	−125.2 (8)	O2^iii^—Sr1—O4—Sr1^iii^	−83.58 (18)
O4^iv^—Sr1^iv^—O2—C1	136.7 (8)	O3—Sr1^iii^—O4—Sr1	9.45 (18)
O5^iv^—Sr1^iv^—O2—C1	64.1 (8)	O2^i^—Sr1^iii^—O4—Sr1	−51.45 (16)
O6^iv^—Sr1^iv^—O2—C1	−48.4 (8)	O1^iii^—Sr1^iii^—O4—Sr1	79.93 (18)
O2^ii^—Sr1^iv^—O2—C1	−174.6 (7)	O3^iii^—Sr1^iii^—O4—Sr1	−128.6 (2)
O3^iv^—Sr1^ii^—O2—C1	127.5 (6)	O4^iii^—Sr1^iii^—O4—Sr1	−58.9 (3)
O4^iv^—Sr1^ii^—O2—C1	−135.0 (6)	O5^iii^—Sr1^iii^—O4—Sr1	53.5 (3)
O1^ii^—Sr1^ii^—O2—C1	175.1 (5)	O6^iii^—Sr1^iii^—O4—Sr1	157.2 (2)
O3^ii^—Sr1^ii^—O2—C1	−50.0 (5)	Sr1—O1—C1—O2	−19.8 (13)
O4^ii^—Sr1^ii^—O2—C1	39.4 (5)	Sr1—O1—C1—C2	158.9 (6)
O5^ii^—Sr1^ii^—O2—C1	95.3 (5)	Sr1^iv^—O2—C1—O1	−172.8 (5)
O6^ii^—Sr1^ii^—O2—C1	−98.7 (5)	Sr1^ii^—O2—C1—O1	13.2 (10)
O2^iii^—Sr1^ii^—O2—C1	−5.0 (6)	Sr1^iv^—O2—C1—C2	8.5 (11)
O1—Sr1—O3—Sr1^iii^	−171.81 (15)	Sr1^ii^—O2—C1—C2	−165.5 (4)
O4—Sr1—O3—Sr1^iii^	9.48 (18)	C2—N1—C3—C4A	−10.6 (17)
O5—Sr1—O3—Sr1^iii^	−36.0 (4)	C3—N1—C2—C5	34.0 (8)
O6—Sr1—O3—Sr1^iii^	−135.3 (2)	C3—N1—C2—C1	160.9 (6)
O2^i^—Sr1—O3—Sr1^iii^	−52.54 (16)	O1—C1—C2—N1	4.6 (9)
O3^ii^—Sr1—O3—Sr1^iii^	80.0 (3)	O2—C1—C2—C5	−58.5 (9)
O4^ii^—Sr1—O3—Sr1^iii^	146.5 (2)	O2—C1—C2—N1	−176.6 (6)
O2^iii^—Sr1—O3—Sr1^iii^	83.01 (17)	O1—C1—C2—C5	122.7 (7)
O4—Sr1^iii^—O3—Sr1	−9.45 (18)	N1—C2—C5—C4A	−43.4 (12)
O2^i^—Sr1^iii^—O3—Sr1	51.95 (16)	C1—C2—C5—C4A	−166.7 (12)
O1^iii^—Sr1^iii^—O3—Sr1	−81.27 (18)	N1—C3—C4A—C5	−17 (2)
O3^iii^—Sr1^iii^—O3—Sr1	55.3 (3)	C3—C4A—C5—C2	37 (2)
O4^iii^—Sr1^iii^—O3—Sr1	127.5 (2)		

Symmetry codes: (i) *x*+1, *y*, *z*; (ii) *x*−1/2, −*y*+1/2, −*z*+1; (iii) *x*+1/2, −*y*+1/2, −*z*+1; (iv) *x*−1, *y*, *z*.

### Hydrogen-bond geometry (Å, º)

**Table:**

*D*—H···*A*	*D*—H	H···*A*	*D*···*A*	*D*—H···*A*
N1—H1*A*···Br2^i^	0.90 (6)	2.52 (5)	3.374 (7)	159 (6)
N1—H1*B*···Br3^i^	0.90 (7)	2.40 (7)	3.240 (7)	156 (8)
O3—H3*C*···Br3^v^	0.84 (7)	2.63 (7)	3.440 (6)	163 (7)
O3—H3*D*···Br2^vi^	0.84 (7)	2.54 (7)	3.376 (6)	172 (5)
O4—H4*E*···Br2^vii^	0.85 (6)	2.47 (7)	3.281 (6)	162 (7)
O4—H4*F*···Br3^viii^	0.83 (6)	2.52 (6)	3.347 (6)	174 (6)
O5—H5*C*···Br2^i^	0.86 (5)	2.54 (5)	3.369 (6)	164 (6)
O5—H5*D*···Br3^viii^	0.84 (6)	2.48 (6)	3.304 (6)	166 (6)
O6—H6*C*···Br2^vi^	0.83 (6)	2.58 (6)	3.393 (6)	167 (5)
O6—H6*D*···Br3^i^	0.85 (7)	2.56 (6)	3.378 (6)	162 (7)

Symmetry codes: (i) *x*+1, *y*, *z*; (v) −*x*+3/2, −*y*+1, *z*+1/2; (vi) −*x*+2, *y*+1/2, −*z*+1/2; (vii) −*x*+3/2, −*y*, *z*+1/2; (viii) −*x*+2, *y*−1/2, −*z*+1/2.
